# Automated prediction of HIV drug resistance from genotype data

**DOI:** 10.1186/s12859-016-1114-6

**Published:** 2016-08-31

**Authors:** ChenHsiang Shen, Xiaxia Yu, Robert W. Harrison, Irene T. Weber

**Affiliations:** 1 Department of Biology, Georgia State University, Atlanta, GA 30303 USA; 2 Department of Computer Science, Georgia State University, Atlanta, GA 30303 USA

**Keywords:** Drug resistance prediction, HIV/AIDS drugs, Encoding structure and sequence, Supervised machine learning, Automation

## Abstract

**Background:**

HIV/AIDS is a serious threat to public health. The emergence of drug resistance mutations diminishes the effectiveness of drug therapy for HIV/AIDS. Developing a computational prediction of drug resistance phenotype will enable efficient and timely selection of the best treatment regimens.

**Results:**

A unified encoding of protein sequence and structure was used as the feature vector for predicting phenotypic resistance from genotype data. Two machine learning algorithms, Random Forest and K-nearest neighbor, were used. The prediction accuracies were examined by five-fold cross-validation on the genotype-phenotype datasets. A supervised machine learning approach for automatic prediction of drug resistance was developed to handle genotype-phenotype datasets of HIV protease (PR) and reverse transcriptase (RT). It predicts the drug resistance phenotype and its relative severity from a query sequence. The accuracy of the classification was higher than 0.973 for eight PR inhibitors and 0.986 for ten RT inhibitors, respectively. The overall cross-validated regression R^2^-values for the severity of drug resistance were 0.772–0.953 for 8 PR inhibitors and 0.773–0.995 for 10 RT inhibitors.

**Conclusions:**

Machine learning using a unified encoding of sequence and protein structure as a feature vector provides an accurate prediction of drug resistance from genotype data. A practical webserver for clinicians has been implemented.

## Background

HIV/AIDS is a pandemic disease caused by human immunodeficiency virus (HIV). In the absence of an effective vaccine for HIV, current treatment of AIDS/HIV patients relies on Highly Active Antiretroviral Therapy (HAART). HAART uses a combination of drugs that target different steps in the viral life cycle to prolong the life of patients. The antiviral drugs, and the structure and mechanism of their targets are reviewed in [1]. The viral enzymes, HIV-1 protease (PR) and reverse transcriptase (RT), are important and well characterized drug targets. The enzymatic activity of these two proteins is blocked by the antiviral PR inhibitors (PIs) and the active site (NRTIs) and non-active site inhibitors (NNRTIs) of RT.

The rapid selection of drug resistant viral mutations raises a challenge for therapy. The presence of these resistance mutations in the infecting virus is an important contraindication for an effective virological response to HAART [2, 3]. At present, genotypic and phenotypic tests are the two major methods for assessing the drug resistance of HIV mutants. The most widely used tool is the genotypic test where the sequence of the viral genome is analyzed for the presence of known drug resistance mutations [4]. In the phenotypic test, the susceptibility to drugs is measured for cells infected with the viral strain in vitro [5]. The phenotypic test directly determines the drug resistance profile of the viral strain, however, it is relatively slower and more expensive than the genotypic test. Ideally, a highly accurate genotypic test would be valuable in the clinic to quickly and inexpensively establish an effective antiretroviral regimen.

**Fig. 1:**
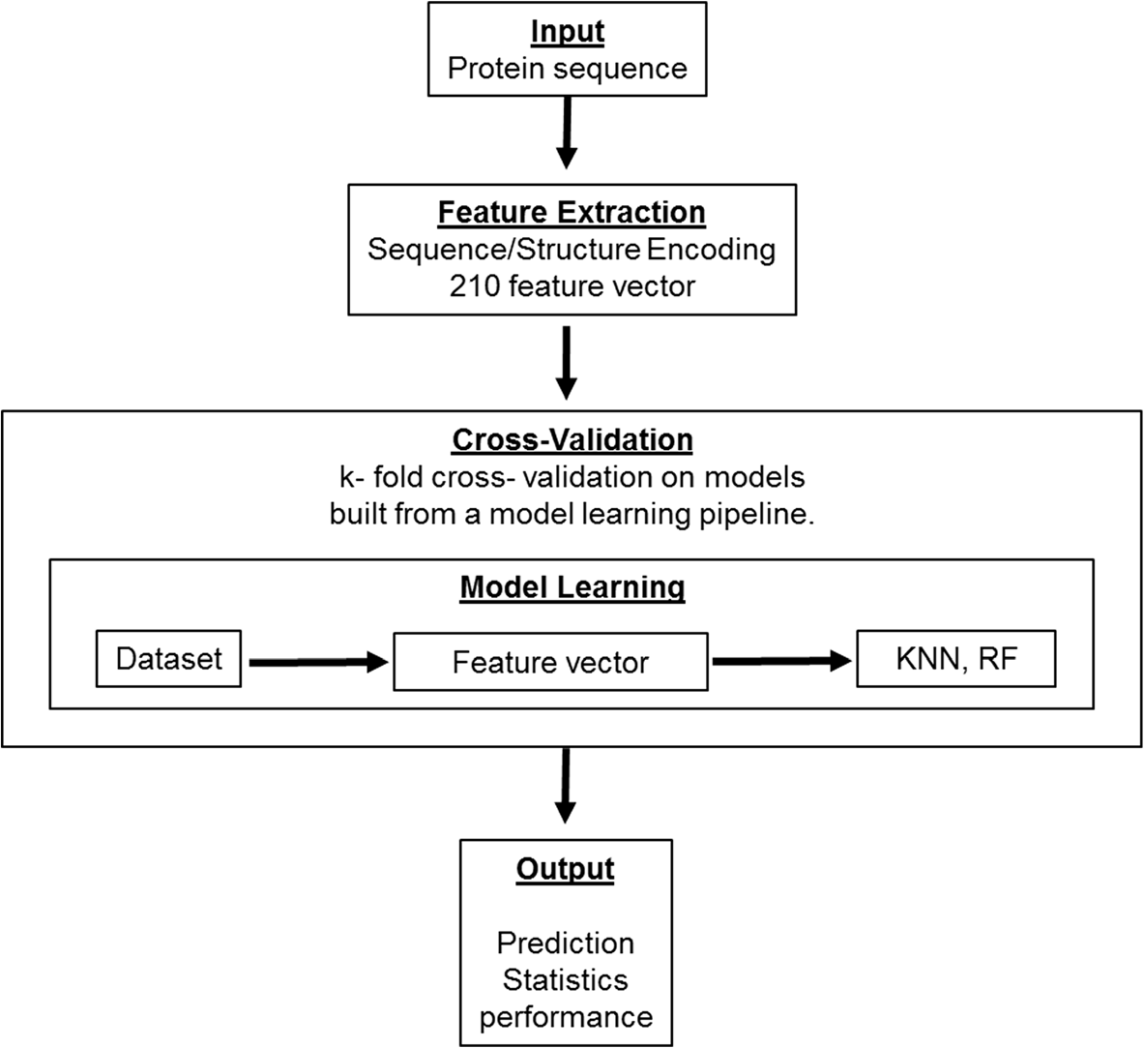
Workflow of prediction server

In principle, drug resistance can be predicted from the presence of specific mutations in the viral genome. The existence of multiple mutations in many different combinations prevents naive direct interpretation of the mutations, and poses a major challenge [6]. Several approaches using machine learning, such as linear regression [7], decision trees [8], neural networks [9], support vector regression [10], and Bayesian networks [11], and rule-based methods, such as Stanford HIVdb [12], HIV-GRADE [13], and ANRS [14], have been proposed for the interpretation of genotypic tests [15]. In our previous studies, we predicted phenotypic results successfully from PR and RT sequences by applying a unified encoding of sequence and protein structure as a feature vector. This approach worked well with several unique machine learning algorithms and obtained significantly higher accuracy than other methods [7, 16]. Our classification accuracies were in the range of 93–99 % vs. 60–85 % for the other methods with HIV protease. The aim of this paper is to develop and implement a phenotype prediction webservice that can be used to guide the selection of drugs to treat people with resistant infections. The service applies the unified sequence/structure encoding and the machine learning algorithms, K-nearest neighbor (KNN) and Random Forest (RF), for HIV genomic data for PR and RT. The overall workflow of the prediction service is shown in Fig. 1 and the webserver is freely available at http://apollo.cs.gsu.edu/~bshen/html/index.html.

Developing a public webservice for drug resistance converts a pure research problem into an applied engineering problem. The machine learning algorithm must be chosen to allow automatic updating as the underlying database acquires more data. We chose the KNN and RF machine learning algorithms because they are reliable in this context. In addition to simply classifying the sequence as resistant/non-resistant, it is critical to predict the relative strength of the resistance in order to select the most effective drug. Therefore the server performs regression as well as classification. The novelty in this work is not as much the choice of machine learning algorithm or encoding, but their combination into an effective and usable webservice.

The service was trained on existing drug specific datasets that are publicly available, and five-fold cross validation was applied to evaluate the quality of the machine learning model. The server accepts amino acid sequences in FASTA-format as query samples. Each sequence is automatically mapped onto the structure and a 210 dimensional feature vector is generated as described in the methods section. The server predicts the phenotype of the query sequence from an online trained machine with KNN and RF. The analysis reported here includes the detailed evaluation of model performance and the overall accuracy of prediction.

## Methods

### Preparation of the datasets

The publicly available high quality filtered datasets were obtained from the HIV drug resistance database, which includes the results of drug susceptibility tests analyzed using the PhenoSense assay and the viral sequences of PR and RT [17] (http://hivdb.stanford.edu/pages/genopheno.dataset.html). Eight PR inhibitors, tazanavir (ATV), indinavir (IDV), nelfinavir (NFV), amprenavir (APV), darunavir (DRV), lopinavir(LPV), tipranavir (TPV) and saquinavir (SQV), were included in the datasets. Ten HIV RT inhibitors (RTIs), including active site (NRTIs) and non-active site inhibitors (NNRTIs), nevirapine (NVP), rilpivirine(RPV), efavirenz (EFV), Etravirine (ETR), lamivudine (3TC), abacavir (ABC), zidovudine (AZT), stavudine (D4T), didanosine (DDI) and tenofovir (TDF), were included in the datasets. The datasets remove redundancies.

The ambiguous mutations in the genotype data were expanded into several individual protein sequences with the same value of drug susceptibility. For example, if there are two residues, both having two different mutations in the original sequence, the total number of sequences after reconstruction will be 4 = (2 × 2) and all will have the same resistance value. The input sequences with insertion or deletion of amino acids were excluded from the expanded datasets because of the lack of structural information for feature extraction.

### Drug susceptibility and cutoffs for resistance/susceptibility

The susceptibility values of each drug reported from the datasets are expressed as the fold change defined as the ratio of the IC_50_ of a mutant and a wild-type standard assayed by the PhenoSense assay. The distribution of phenotypic results affects the accuracy of classification. There is a specific cutoff for each drug that determines when the virus is considered resistant to that drug. Cutoff values were obtained from [18–20]. If the resistance is less than the cutoff value, the mutant is classified as non-resistant or susceptible to the drug, and reported as 0. Otherwise, the mutant is considered as drug resistant, and reported as 1. The cutoff values for PIs were as follows: 2.0 for TPV, 3.0 for NFV, SQV, IDV, and ATV, 4.0 for FPV, 9.0 for LPV, and 10.0 for DRV. For NRTIs, the cutoff values were: 1.5 for D4T, TDF, and DDI, and 3.0 for AZT, ABC and 3TC. For the NNRTIs the cutoff was 3.0.

### Feature representation

Crystal structures of HIV PR and RT (pdb id: 2wom and 3oxc) were downloaded from the PDB (Protein Data Bank at http://www.rcsb.org/pdb/) to serve as the structural templates. Delaunay triangulation was applied to extract a subset of interactions from the Cα atoms [21]. This triangulation defines a graph of pairs of residues that have direct interactions in space and thus summarizes the protein structure. The triangulation removes the dependence of the representation on the origin and orientation of the protein molecule. There are 210 unique pairs of the twenty different amino acids in proteins. The feature vector was determined by summing the distances between Cα atoms along each arc of the Delaunay triangulation where the arc connected two amino acids of the given type. For example, if an arc connected an Alanine(A) to a Phenylalanine(F), then the element of the feature vector for an (A,F) pair had the distance between that pair of residues added to it. Summarizing the content of the arcs into a 210 element vector results in a compact and efficient representation of both the sequence and the structure. Compared to the calculation for all residue pairs, the application of the unified sequence/structure encoding in feature extraction successfully removed noise and indirect connections of residue pairs from the vector and reduced the size of features, thus improving the performance of the learning/prediction process [21].

### Supervised machine learning

The K-nearest neighbor and Random Forest algorithms were used to train a learning model from the 210 dimension vectors of training samples paired with phenotypic data. The phenotype of the testing samples was predicted from learning model. The output from the learning model is a discrete label for the classification of the phenotype and is a continuous number for the regression analysis of relative resistance.

The KNN algorithm is a non-parametric method that uses the full training data set. It finds the K nearest neighbors to a query point and reports either their class by majority vote or the average of their resistance value. K was set to 6 for both classification and regression. The benefit of applying KNN is that the training stage is faster, but unlike SVM and sparse dictionary, KNN uses the complete training data in the prediction stage because the result is reported based on the training data. Updating a KNN predictor with new experimental resistance data is especially straightforward and simply requires performing the feature extraction step on the new data.

The RF algorithm is an ensemble based classifier/regression that works with multiple decision trees to improve the accuracy. The disadvantage of individual decision trees is that they are sensitive to small changes of selected features in the training space. Therefore an individual decision tree is a weak learner with a poor ability to generalize and a strong tendency to be unstable. RF uses the ensemble votes from multiple decision trees to improve the stability of trained machine as well as the prediction accuracy. In practice, the RF algorithm calculates the averaged value voted from different sub-trees that randomly built from the training dataset. The number of sub-trees is set to 10, and the criterion for the quality of split is the mean squared error.

### Cross validation

Cross-validation with 5 random folds was applied for all classifier and regression analysis to assess the quality of the machine learning models. Our previous work [7] showed that 5-fold cross validation was an appropriate statistical measure of quality for this dataset. All the sequences, including drug resistant and non-drug resistant mutants, were randomly assigned to one of five sets. For each cross-validation one of the five sets was reserved for testing and the other four used to train the machine. At the end of validation, the average error across the 5 tests was calculated. For classification, the accuracy (Eq. 1), sensitivity (Eq. 2) and specificity (Eq. 3) were calculated, and regression reported R^2^ value.

1 $$ \mathrm{Accuracy} = \left[\mathrm{T}\mathrm{P} + \mathrm{T}\mathrm{N}\right]/\left[\mathrm{T}\mathrm{P} + \mathrm{F}\mathrm{N} + \mathrm{T}\mathrm{N} + \mathrm{F}\mathrm{P}\right] $$

2 $$ \mathrm{Sensitivity} = \mathrm{T}\mathrm{P}/\left[\mathrm{T}\mathrm{P} + \mathrm{F}\mathrm{N}\right] $$

3 $$ \mathrm{Specificity} = \mathrm{T}\mathrm{N}/\left[\mathrm{T}\mathrm{N} + \mathrm{F}\mathrm{P}\right] $$

Where TP, TF, FP, and FN are the true positive, true negative, false positive and false negative, respectively.

### Implementation and run-time analyses

The automated prediction server was implemented with the Python programming language (v. 2.7.6). Open sourced libraries, BioPython (v. 1.65) for parsing sequence data, SciPy (scipy.org, v.0.15.0) for Delaunay triangulation, NumPy (numpy.org, v. 1.9.1) for vector operations; and scikit-learn (scikit-learn.org, v. 0.16.1) for different machine-learning algorithms, were applied to construct the system. The application architecture consists of the client front end, HTML and shell-based processes, PHP and Python, for analysis. Tests were performed on a DELL PRECISION T5500 with two Intel® Xeon E5607 CPU, 8 cores available, and 24 GB RAM running Ubuntu 10.04.

## Results

### Datasets

The 210 dimensional vectors were constructed from the genotype-phenotype datasets for classification and regression analysis. The details of the preprocessing of the sequence and resistance values are described in Methods. After the expansion of genotype data to unique protein sequences, there were 11,314 to 13,795 unique sequences of HIV PR mutants and 4,540 to 259,347 sequences of RT mutants for the various resistance values to the inhibitors. The reconstructed sequences of PR include wild type and mutants with a maximum of 31 substitutions. For RT datasets, the reconstructed datasets contain wild type and mutants with a maximum of 35 mutations. The expanded datasets were used for the model learning and validation of learned models.

### Regression on resistance to inhibitors of PR and RT

The KNN and RF regression analyses were performed on genotype-phenotype data for HIV PR and RT to predict the resistance values of the query samples. The R^2^ values are listed in Tables 1, 2 and 3 as the average of all the R^2^ values from the 5-fold analysis. The analysis for eight PR inhibitors gave R^2^ values of 0.719–0.928 for KNN regression and 0.772–0.953 for RF (Table 1). Both KNN and RF regressions gave high R^2^ values with standard deviation lower than 0.05 for all PIs, except for TPV. In comparison, we reported lower R^2^ values of 0.579–0.783 for multi linear regression on seven PIs using a smaller dataset [7]. For six NRTIs, RF regression gave R^2^ values of 0.883–0.989 and KNN regression gave R^2^ values of 0.816–0.986 (Table 2), again showing an improvement over the earlier results of 0.614–0.975 for multi linear regression on six NRTIs. Even higher R^2^ values of 0.937–0.995 for RF regression and 0.895–0.980 for KNN regression were obtained for four NNRTIs (Table 3). The previous results for three NNRTIs gave R^2^ values of 0.850–0.904 for multi linear regression. Thus, both KNN and RF regressions improve the R^2^ values and show reasonable standard deviations over calculations with multi linear regression. Therefore, the graph based encoding with regression had outstanding predictions of resistance to eight PR inhibitors and ten RT inhibitors.

**Table 1:** Regression on predicted resistance for eight PR inhibitors

	RF Regression		KNN Regression	
	R^2^ values		R^2^ values	
	mean	stddev	mean	stddev
SQV	0.858	0.034	0.719	0.042
LPV	0.953	0.010	0.928	0.013
FPV	0.859	0.027	0.822	0.032
DRV	0.920	0.019	0.924	0.019
ATV	0.906	0.016	0.851	0.032
NFV	0.909	0.026	0.836	0.020
TPV	0.772	0.147	0.735	0.102
IDV	0.890	0.023	0.794	0.045

**Table 2:** Regression on predicted resistance for six NRTIs

	RF Regression		KNN Regression	
	R^2^ values		R^2^ values	
	mean	stddev	mean	stddev
AZT	0.881	0.040	0.816	0.051
DDI	0.924	0.116	0.960	0.011
D4T	0.974	0.023	0.918	0.055
3TC	0.989	0.002	0.968	0.002
ABC	0.773	0.188	0.785	0.135
TDF	0.964	0.008	0.903	0.034

**Table 3:** Regression on predicted resistance for four NNRTIs

	RF Regression		KNN Regression	
	R^2^ values		R^2^ values	
	mean	stddev	mean	stddev
EFV	0.985	0.008	0.980	0.009
NVP	0.995	0.001	0.986	0.001
ETR	0.955	0.022	0.929	0.020
RPV	0.937	0.022	0.895	0.044

### Classification using k-nearest neighbor

KNN algorithm is widely used as a supervised learning classifier for the machine learning classification. Five-fold cross validation tests were performed, the results are shown in Tables 4, 5, and 6 for HIV-1 PIs, HIV RT NRTIs, and NNRTIs, respectively. Using KNN shows high values of accuracy, sensitivity and specificity. For classification of resistance of protease inhibitors, the values calculated for accuracy, sensitivity and specificity have a low of 0.963 and a high of 0.99. Resistance to NRTIs is classified with accuracies of 0.986–0.991, sensitivities of greater than 0.984 and specificities of greater than 0.986, while for NNRTIs the classification was superior showing values over 0.983 for accuracy, sensitivity and specificity. The run times of 5-fold validation with KNN ranged from 5.1 to 1283.7 s.

**Table 4:** Classification using KNN for resistance to PIs

	SQV	LPV	FPV	DRV	ATV	NFV	TPV	IDV
Accuracy	0.973	0.979	0.971	0.989	0.982	0.981	0.985	0.979
stddev	0.003	0.003	0.005	0.003	0.002	0.001	0.002	0.002
Sensitivity	0.965	0.977	0.963	0.988	0.979	0.976	0.986	0.976
stddev	0.005	0.004	0.008	0.005	0.005	0.002	0.004	0.002
Specificity	0.980	0.981	0.980	0.990	0.986	0.985	0.984	0.982
stddev	0.004	0.003	0.005	0.004	0.002	0.002	0.003	0.005
Run time	17.2	18.3	21.0	5.1	18.5	31.8	8.8	26.4

**Table 5:** Classification using KNN for resistance to NRTIs

	AZT	DDI	D4T	3TC	ABC	TDF
Accuracy	0.988	0.989	0.991	0.992	0.990	0.986
stddev	0.002	0.001	0.001	0.001	0.001	0.002
Sensitivity	0.984	0.986	0.989	0.988	0.988	0.985
stddev	0.003	0.001	0.002	0.002	0.001	0.002
Specificity	0.991	0.991	0.993	0.995	0.991	0.986
stddev	0.002	0.001	0.001	0.001	0.002	0.003
Run time	98.5	142.7	144.7	143.1	166.3	56.1

**Table 6:** Classification using KNN for resistance to NNRTIs

	EFV	NVP	RPV	ETR
Accuracy	0.996	0.996	0.987	0.995
stddev	0.000	0.000	0.001	0.001
Sensitivity	0.996	0.995	0.983	0.992
stddev	0.000	0.001	0.003	0.002
Specificity	0.997	0.997	0.992	0.997
stddev	0.000	0.001	0.003	0.001
Run time	1199.8	1283.7	7.2	48.9

### Classification using random forest

The predicted and observed phenotype were compared and the results are shown in Tables 7, 8 and 9 for HIV PIs, RT NRTIs and NNRTIs, respectively. RF classification provides superior values for accuracy, sensitivity and specificity for all PR and RT inhibitors. Resistance to protease inhibitors was classified with the values for accuracy, sensitivity and specificity calculated at 0.98–0.99. Resistance to NRTIs is classified with values of greater than 0.99 for accuracy, sensitivity and specificity, while for NNRTIs the classification performance also achieved values of over 0.985. We recorded the run time of 5-fold validation with RF classifier, the performance ranged from 2.2 to 69.3 s for 10 RT inhibitors.

**Table 7:** Classification using RF for resistance to PIs

	SQV	LPV	FPV	DRV	ATV	NFV	TPV	IDV
Accuracy	0.984	0.988	0.981	0.992	0.986	0.988	0.988	0.989
stddev	0.002	0.003	0.003	0.004	0.002	0.002	0.004	0.001
Sensitivity	0.983	0.986	0.977	0.993	0.988	0.987	0.987	0.987
stddev	0.002	0.004	0.005	0.004	0.005	0.004	0.007	0.003
Specificity	0.986	0.989	0.984	0.992	0.984	0.99	0.988	0.99
stddev	0.003	0.004	0.001	0.004	0.002	0.003	0.002	0.002
Run time	3.6	3.8	4.0	2.2	4	4.6	2.9	4.3

**Table 8:** Classification using RF for resistance to NRTIs

	AZT	DDI	D4T	3TC	ABC	TDF
Accuracy	0.994	0.993	0.994	0.997	0.994	0.992
stddev	0.001	0.001	0.001	0.001	0.000	0.001
Sensitivity	0.994	0.993	0.993	0.997	0.994	0.99
stddev	0.002	0.001	0.002	0.001	0.001	0.003
Specificity	0.995	0.993	0.994	0.997	0.994	0.993
stddev	0.001	0.002	0.001	0.001	0.001	0.002
Run time	8.9	13.6	12.2	9.7	10.7	6.6

**Table 9:** Classification using RF for resistance to NNRTIs

	EFV	NVP	RPV	ETR
Accuracy	0.998	0.998	0.989	0.997
stddev	0.000	0.000	0.003	0.000
Sensitivity	0.998	0.998	0.985	0.995
stddev	0.000	0.001	0.006	0.001
Specificity	0.998	0.998	0.993	0.998
stddev	0.000	0.000	0.002	0.000
Run time	67.8	69.3	3.7	8.0

The KNN classification algorithm is capable of handling large volumes of data in near real-time which makes it eminently suitable for deployment in an automated webservice [22]. In our tests, the KNN and RF classifications provide higher accuracy compared to our previous results where the values for accuracy were calculated in the range of 0.93–0.99 for SVM and ANN classifications [7], as well as improved R^2^ values from regression analyses. These results suggest both algorithms perform well with the high dimensional data and a large number of training examples.

## Discussion

This unified sequence-structure encoding gave high accuracy in initial tests on four PIs [16] and subsequent expansion to seven inhibitors of HIV PR and nine inhibitors of RT [7]. Here, we used an expanded data set, which included more genotype/phenotype data and additional data for two drugs, darunavir and amprenavir. We also evaluated machine learning tools with implementations that are compatible with web services. Regression and classification analysis on resistance data were performed for eight inhibitors of HIV PR and ten inhibitors of RT. Both KNN and RF regressions provide better R^2^ values than the multi-linear regression applied in our previous study [7]. The lower R^2^ values obtained from multi-linear regression may occur because the structural effects induced by the multiple mutations are not interacting linearly. Each single mutation can have various effects on the overall function of the protein, such as altering the interactions between the protein and its inhibitor, altering the catalytic activity and changing the stability of the protein, however, the mutations accumulate in different combinations to produce higher level resistance, which makes it difficult to interpret phenotypic data though linear regression. In the case of HIV PR, different sets of about 20 mutations produce high level resistance by altering the structure, activity and inhibition as reviewed in [23].

Classification with KNN or RF methods also had high accuracies for predicting the drug resistance for PR and RT inhibitors. Importantly, both algorithms can reliably predict the phenotype of an unknown sample because the prediction of query sample relies on how well the features match with the training sample. One weakness of applying KNN or RF regression is that the interpreted phenotypic values cannot exceed the cutoff values obtained from the experiment in the training space.

The current implementation of the encoding scheme did not handle deletions or insertions in the protein sequence. Another group applied a normalized protein sequence to extract features for the machine learning [24]. A future direction for this research is to expand the representation to handle insertions and deletions using normalization techniques similar to those explored in our previous work [21] for proteins of varying sizes.

## Conclusions

Our unified encoding of protein sequence and structure using Delaunay triangulation results in a unique 210 element vector for each protein, which is a compact and efficient representation. The application of the unified encoding as a feature vector for machine learning provides an accurate prediction of HIV drug resistance from genotype data. This approach has been implemented in a practical webservice. The webserver for predicting resistance is freely available at http://apollo.cs.gsu.edu/~bshen/html/index.html.

## References

[1] Menendez-Arias L (2013). Molecular basis of human immunodeficiency virus type 1 drug resistance: overview and recent developments. Antiviral Res.

[2] Saracino A, Monno L, Locaputo S, Torti C, Scudeller L, Ladisa N, Antinori A, Sighinolfi L, Chirianni A, Mazzotta F (2004). Selection of antiretroviral therapy guided by genotypic or phenotypic resistance testing: an open-label, randomized, multicenter study (PhenGen). J Acquir Immune Defic Syndr.

[3] Descamps D, Brun-Vezinet F, Geretti AM (2006). Benefits of resistance testing.

[4] Durant J, Clevenbergh P, Halfon P, Delgiudice P, Porsin S, Simonet P, Montagne N, Boucher CA, Schapiro JM, Dellamonica P (1999). Drug-resistance genotyping in HIV-1 therapy: the VIRADAPT randomised controlled trial. Lancet.

[5] Cohen CJ, Hunt S, Sension M, Farthing C, Conant M, Jacobson S, Nadler J, Verbiest W, Hertogs K, Ames M (2002). A randomized trial assessing the impact of phenotypic resistance testing on antiretroviral therapy. AIDS.

[6] Schutten M, Geretti AM (2006). Resistance assays.

[7] Yu X, Weber IT, Harrison RW (2014). Prediction of HIV drug resistance from genotype with encoded three-dimensional protein structure. BMC Genomics.

[8] Beerenwinkel N, Schmidt B, Walter H, Kaiser R, Lengauer T, Hoffmann D, Korn K, Selbig J (2002). Diversity and complexity of HIV-1 drug resistance: a bioinformatics approach to predicting phenotype from genotype. Proc Natl Acad Sci U S A.

[9] Wang D, Larder B (2003). Enhanced prediction of lopinavir resistance from genotype by use of artificial neural networks. J Infect Dis.

[10] Beerenwinkel N, Daumer M, Oette M, Korn K, Hoffmann D, Kaiser R, Lengauer T, Selbig J, Walter H (2003). Geno2pheno: estimating phenotypic drug resistance from HIV-1 genotypes. Nucleic Acids Res.

[11] Deforche K, Silander T, Camacho R, Grossman Z, Soares MA, Van Laethem K, Kantor R, Moreau Y, Vandamme AM, non BW (2006). Analysis of HIV-1 pol sequences using Bayesian Networks: implications for drug resistance. Bioinformatics.

[12] Liu TF, Shafer RW (2006). Web resources for HIV type 1 genotypic-resistance test interpretation. Clin Infect Dis.

[13] Obermeier M, Pironti A, Berg T, Braun P, Daumer M, Eberle J, Ehret R, Kaiser R, Kleinkauf N, Korn K (2012). HIV-GRADE: a publicly available, rules-based drug resistance interpretation algorithm integrating bioinformatic knowledge. Intervirology.

[14] Brun-Vezinet F, Descamps D, Ruffault A, Masquelier B, Calvez V, Peytavin G, Telles F, Morand-Joubert L, Meynard JL, Vray M (2003). Clinically relevant interpretation of genotype for resistance to abacavir. AIDS.

[15] Humphris-Narayanan E, Akiva E, Varela R, Ó Conchúir S, Kortemme T (2012). Prediction of mutational tolerance in HIV-1 protease and reverse transcriptase using flexible backbone protein design. PLoS Comput Biol.

[16] Yu X, Weber IT, Harrison RW (2013). Sparse representation for prediction of HIV-1 protease drug resistance. Proc SIAM Int Conf Data Min.

[17] Rhee SY, Gonzales MJ, Kantor R, Betts BJ, Ravela J, Shafer RW (2003). Human immunodeficiency virus reverse transcriptase and protease sequence database. Nucleic Acids Res.

[18] Rhee SY, Taylor J, Fessel WJ, Kaufman D, Towner W, Troia P, Ruane P, Hellinger J, Shirvani V, Zolopa A (2010). HIV-1 protease mutations and protease inhibitor cross-resistance. Antimicrob Agents Chemother.

[19] Melikian GL, Rhee SY, Taylor J, Fessel WJ, Kaufman D, Towner W, Troia-Cancio PV, Zolopa A, Robbins GK, Kagan R (2012). Standardized comparison of the relative impacts of HIV-1 reverse transcriptase (RT) mutations on nucleoside RT inhibitor susceptibility. Antimicrob Agents Chemother.

[20] Rhee SY, Liu T, Ravela J, Gonzales MJ, Shafer RW (2004). Distribution of human immunodeficiency virus type 1 protease and reverse transcriptase mutation patterns in 4,183 persons undergoing genotypic resistance testing. Antimicrob Agents Chemother.

[21] Bose P, Xiaxia Y, Harrison RW (2011). Encoding protein structure with functions on graphs. Bioinformatics and Biomedicine Workshops (BIBMW), 2011 IEEE International Conference on: 12–15 Nov. 2011.

[22] Adeniyi DA, Wei Z, Yongquan Y. Automated web usage data mining and recommendation system using K-Nearest Neighbor (KNN) classification method. Applied Computing and Informatics. 2015. http://dx.doi.org/10.1016/j.aci.2014.10.001.

[23] Weber IT, Kneller DW, Wong-Sam A (2015). Highly resistant HIV-1 proteases and strategies for their inhibition. Future medicinal chemistry.

[24] Heider D, Verheyen J, Hoffmann D (2011). Machine learning on normalized protein sequences. BMC research notes.

